# BMI‐dependent prognostic role of EEF1G in breast cancer: A 15‐year follow‐up of the Guangzhou Breast Cancer Cohort Study

**DOI:** 10.1002/cam4.70227

**Published:** 2025-09-09

**Authors:** Na Li, Chengkun Xiao, Shushu Han, Minjie Lu, Qianxin Chen, Yuanzhong Yang, Luying Tang, Zefang Ren, Lin Xu

**Affiliations:** ^1^ School of Public Health Sun Yat‐sen University Guangzhou China; ^2^ The Sun Yat‐Sen University Cancer Center Guangzhou China; ^3^ The Third Affiliated Hospital Sun Yat‐Sen University Guangzhou China; ^4^ School of Public Health The University of Hong Kong Hong Kong China; ^5^ Institute of Applied Health Research, University of Birmingham Birmingham UK

**Keywords:** BMI, breast cancer, EEF1G, prognosis

## Abstract

**Objective:**

Eukaryotic elongation factor 1 gamma (EEF1G) has emerged as a potential prognostic marker in various malignancies. Yet, its association with breast cancer (BC) prognosis, particularly in the context of body mass index (BMI) status, remains unexplored. Therefore, we investigated the prognostic value and role of EEF1G in BC across different BMI categories.

**Methods:**

EEF1G expression was assessed through immunohistochemistry in tissue microarrays on 1011 patients with primary invasive BC. Prognostic effects were analyzed using the Cox proportional hazards regression. GSE78958 dataset downloaded from the Gene Expression Omnibus (GEO) database was used to validate our findings. Gene Set Enrichment Analysis (GSEA) was performed using R packages, and protein–protein interaction (PPI) networks were generated using the STRING database and Cytoscape software.

**Results:**

Elevated EEF1G expression was associated with a better prognosis in patients with BMI ≤ 24 kg/m^2^ (hazard ratio (HR) for overall mortality = 0.67, 95% confidence interval (CI): 0.43–1.03; HR for progression = 0.60, 95% CI: 0.42–0.86). In contrast, for patients with BMI > 24 kg/m^2^, it appeared to be associated with poorer outcomes (HR for overall mortality = 1.74, 95% CI: 0.96–3.17; HR for progression = 1.63, 95% CI: 1.00–2.66). In patients with BMI > 24 kg/m^2^, EEF1G was associated with specific metabolic and oncogenic pathways, which were not statistically significant in patients with BMI ≤ 24 kg/m^2^. The top interacting genes with *EEF1G* differed between the BMI categories.

**Conclusions:**

This study showed EEF1G expression was inversely associated with BC prognosis in different BMI categories, indicating its potential as a prognostic marker and therapeutic target in BC. The differential effects underscore the need for personalized approaches in BC management and research.

## 1 INTRODUCTION

Breast cancer (BC) is a major global health challenge, being one of the most prevalent and life‐threatening disease affecting women worldwide.^1^, ^2^ Despite significant advancements in diagnostic and therapeutic strategies, the prediction of patient outcomes in BC remains a complex challenge.^3^, ^4^, ^5^ Hence, identifying prognostic factors is crucial for tailoring personalized treatment strategies in BC.

One promising candidate for such a prognostic factor is eukaryotic elongation factor 1 gamma (EEF1G). EEF1G is a constituent of Eukaryotic Elongation Factor complex 1 (EEF1), which is pivotal in protein synthesis, specifically in the transfer of aminoacyl‐tRNA to ribosomes during protein elongation.^6^, ^7^ Abnormal EEF1G expression has been implicated in oncogenic activities across diverse cancer types, suggesting its potential as both a diagnostic and prognostic marker. Notably, a study showed the role of oncogene‐induced ubiquitination of EEF1G in tumor suppression, highlighting its function in mitigating uncontrolled cellular proliferation due to aberrant oncogenic signaling.^8^ Additionally, another study showed how the co‐upregulation of EEF1G and TRAP1 contributed to the synthesis of stress‐responsive proteins, an important adaptive mechanism enabling cancer cells to counteract antitumor therapies.^9^

To date, limited study has specifically investigated the association between EEF1G and BC prognosis. A study using the public platform Kaplan–Meier plotter (http://kmplot.com/analysis/) explored this association.^10^ However, it did not account for confounding factors. Moreover, existing literature indicates a differential expression and functionality of EEF1G in adipocytes derived from lean and obese individuals,^11^ suggesting that body mass index (BMI) may modulate the prognostic relevance and biological effect of EEF1G in BC.

Therefore, our study aims to investigate the prognostic value of EEF1G in BC with a particular focus on its interaction with BMI status. Understanding the differential pathways and gene interactions mediated by EEF1G in lean versus obese patients could provide evidence for identifying novel therapeutic targets.

## 2 MATERIALS AND METHODS

### 2.1 Study population

EEF1G expression was assessed in tissue microarrays (TMAs) obtained from a subset of the Guangzhou Breast Cancer Study (GZBCS) cohort. The cohort comprised 1063 female patients with pathologically diagnosed primary invasive BC, each presenting with tumor sizes exceeding 1 cm in diameter. Patient recruitment spanned from January 2008 to December 2015 at the Cancer Center of Sun Yat‐sen University in Guangzhou, China. We excluded 34 patients lacking complete EEF1G data, yielding a final cohort of 1011 patients (98.3% follow‐up rate) with comprehensive records until December 31, 2022. Ethical approval was granted by the ethics committee of the School of Public Health, Sun Yat‐sen University (IRB No.: 2012–8), and written informed consent was obtained from all participants.

To validate our findings, we used the GSE78958 dataset from the Gene Expression Omnibus (GEO) database (https://www.ncbi.nlm.nih.gov/), which includes mRNA data of 424 BC patients and does not include healthy controls. This dataset encompassed variables such as BMI, ethnicity, histological grade, tumor subtype, and clinical stage, but lacks outcome data (Table S1). After removing 20 samples with missing BMI information, 404 BC patients were included for downstream analyses.

### 2.2 Baseline data collection and follow‐up

Demographic data including age and menopausal status were collected at diagnosis through structured questionnaire‐based interviews conducted by trained research assistants. BMI and clinicopathologic characteristics including clinical stage, histological grade, estrogen receptor (ER), progesterone receptor (PR), human epidermal growth factor receptor 2 (HER‐2) status, and the proliferation index factor Ki‐67, were extracted from medical records.

Patient follow‐up was conducted through regular telephone contacts or outpatient visits. The schedule involved quarterly contacts in the first year, biannual in the second and third years, and yearly thereafter. The primary endpoints of the study were overall survival (OS) and progression‐free survival (PFS). OS was defined as the time from diagnosis to death, while PFS was defined as the time from diagnosis to disease progression, whichever occurred first. Disease progression included recurrence, metastasis, and death.

### 2.3 Tissue microarray and immunohistochemistry

The construction of TMAs was performed as previously described.^12^ The TMAs were subjected to a series of preparatory steps, including baking at 60°C for 2 h, dewaxing with xylene, and rehydration through graded ethanol to distilled water for 10 min. Antigen retrieval was conducted using EDTA buffer (PH 9.0) in a superheated pressure kettle. Endogenous peroxidase activity was quenched using 3% H_2_O_2_. Following this, slides were incubated with rabbit polyclonal antibody (EPR7200) targeting EEF1G (ab124994, diluted 1:2000, Abcam, UK). Detection was performed using the EnVision Detection System (Peroxidase/DAB, Rabbit/Mouse) (Dako K5007, Denmark). Visualization was achieved with diaminobenzidine (DAB) and counterstaining with hematoxylin. Finally, slides were dehydrated and mounted for analysis.

Digital imaging of the immunohistochemically (IHC) stained TMAs was conducted using a Pannoramic Scanner and CaseViewer software (3DHISTECH Ltd., Hungary). An experienced pathologist, who was blinded to the clinical data, evaluated the IHC staining. Staining intensity was scored on a scale from 0 to 3 (0 = no staining, 1 = weak, 2 = moderate, 3 = strong), and the percentage of tumor cell nuclear staining was quantified (ranging from 0% to 100%). The H score was calculated by multiplying the staining intensity by the percentage of tumor cell nuclear staining, yielding a range from 0 to 300. To ensure consistency, the mean the H score from duplicate cores was determined for each case.

### 2.4 GSEA analysis

Gene set enrichment analysis (GSEA) was used to explore the signaling pathways associated with EEF1G expression in BC, stratified by BMI. First, GSEA required the ranking of genes based on their differential expression between groups with low and high EEF1G mRNA expression. This ranking was achieved using statistical methods such as *t*‐tests or fold change analysis, to determine the differential expression levels. Subsequently, GSEA assessed whether genes within predefined sets were predominantly located at the top or bottom of the ranked list. This assessment was facilitated by computing an enrichment score (ES). Following the determination of ES, a statistical test was performed to calculate the significance of each gene set, yielding *p*‐values. These *p*‐values were then adjusted for multiple testing, converting them into False Discovery Rate (FDR) corrected values (normalized ES or NES values) to enhance the robustness of the findings. The GSEA process was conducted using various R packages (version 4.3.0) including “limma”, “org.Hs.eg.db”, “clusterProfiler”, and “pathview.”^13^, ^14^

### 2.5 Construction of protein–protein interaction (PPI) network

Differentially expressed genes were identified using the R “limma” package (version 4.3.0) with selection criterion of “fold change >1” and “adjusted/*p* value ≤ 0.05.” The PPI data were then obtained by inputting the differential genes into the online STRING databases (http://string‐db.org).^15^ When extracting PPI data, we selected a confidence score of 0.4. This resulted in 54 interactions being identified in obese patients and 1300 interactions in nonobese patients for further study. Cytoscape software (version 3.10.1) for additional analyses was used to construct the PPI network.^16^ The plug‐in cytoHubba of Cytoscape was applied to identify and highlight key genes interacting with EEF1G. This approach allowed for the systematic exploration of the interactive landscape surrounding *EEF1G*, providing insights into its potential functional roles and interactions in the context of BC.

### 2.6 Statistical analysis

The median value of both the H‐score and mRNA expression levels of EEF1G was used as the threshold for categorizing patients in to low and high EEF1G expression groups. The associations between EEF1G expression levels and various clinicopathologic parameters were evaluated using the Kruskal–Wallis test and Mann–Whitney *U* test. The Cox proportional hazards regression model was applied to estimate hazard ratios (HR) and 95% confidence interval (CI). Stratified analyses were performed to assess whether the associations between EEF1G expression and BC prognosis were modified by BMI status. An interaction term, created on a multiplicative scale, was included in the model to assess the potential modifying effect of BMI status on the prognosis impact of EEF1G. To explore correlation between EEF1G expression and other genes, Wilcoxon signed‐rank tests and Spearman's rank correlation tests were used. All analyses were conducted using R software (version 4.3.0). Statistical significance was set at a two‐sided *p*‐value of less than 0.05.

## 3 RESULTS

Table 1 shows that most patients were aged 40–59 years (66.2%), premenopausal (58.8%), and with a BMI ≤ 24 kg/m^2^ (63.0%). Most patients were characterized by low histological grade (73.3%), ER positive (73.3%), PR positive (72.7%), HER2 negative (67.2%), and Ki‐67 > 14% (70.7%). Patients with high EEF1G showed more advanced histological grade and lower proportion of ER positive (*p* = 0.032 and 0.011, respectively). No statistically significant differences were observed between the EEF1G expression groups in terms of age at diagnosis, menopausal status, BMI, tumor size, nodal status, tumor subtype, clinical stage, PR status, HER‐2 status, and Ki‐67.

**TABLE 1:** Demographic and clinicopathological characteristics by EEF1G expression groups.

Characteristics	Total, *N* (%)	EEF1G expression, *N* (%)		*p* Value^a^
		Low	High	
Age (years)				
<40	201 (19.9)	105 (19.9)	96 (20.0)	0.51
40–59	667 (66.2)	350 (66.4)	317 (65.9)	
≥60	140 (13.9)	72 (13.7)	68 (14.1)	
Missing	3	3	0	
Menopausal status				
Premenopausal	565 (58.8)	305 (59.8)	260 (57.6)	0.50
Postmenopausal	396 (41.2)	205 (40.2)	191 (42.4)	
Missing	50	20	30	
BMI (kg/m^2^)				
≤24	602 (63.0)	311 (62.6)	291 (64.0)	0.57
>24	353 (37.0)	189 (37.8)	164 (36.0)	
Missing	56	30	26	
Histological grade				
I/II	674 (73.3)	362 (76.4)	312 (70.1)	**0.032**
III	245 (26.7)	112 (23.6)	133 (29.9)	
Missing	92	56	36	
Tumor size (cm)				
≤2	295 (30.5)	160 (31.7)	135 (29.2)	0.39
>2	673 (69.5)	345 (68.3)	328 (70.8)	
Missing	43	25	18	
Nodal status				
Negative	437 (45.1)	220 (43.5)	217 (46.9)	0.29
Positive	532 (54.9)	286 (56.5)	246 (53.1)	
Missing	42	24	18	
Clinical stage				
I/II	701 (71.9)	355 (69.6)	346 (74.4)	0.096
III	274 (28.1)	155 (30.4)	119 (25.6)	
Missing	36	20	16	
Tumor subtype				
Luminal A	336 (39.3)	175 (39.4)	161 (39.3)	0.24
Luminal B	283 (33.1)	158 (35.6)	125 (30.5)	
HER‐2 rich	128 (15.0)	63 (14.2)	65 (15.9)	
Basal‐like	107 (12.5)	48 (10.8)	59 (14.4)	
Missing	157	86	71	
ER status				
Negative	257 (26.7)	117 (23.3)	140 (30.5)	**0.011**
Positive	705 (73.3)	386 (76.7)	319 (69.5)	
Missing	49	27	18	
PR status				
Negative	262 (27.3)	137 (27.3)	125 (27.2)	0.97
Positive	698 (72.7)	364 (72.7)	334 (72.8)	
Missing	51	29	22	
HER‐2 status				
Negative	679 (67.2)	354 (66.8)	325 (67.6)	0.94
Equivocal	85 (8.5)	46 (8.7)	39 (8.1)	
Positive	247 (24.4)	130 (24.5)	117 (24.3)	
Missing	0	0	0	
Ki‐67				
≤14%	223 (29.3)	137 (31.6)	86 (26.3)	0.11
>14%	537 (70.7)	296 (68.4)	241 (73.7)	
Missing	251	97	154	

*Note*: Bold indicates statistically significant values.

Abbreviations: BMI, body mass index; EEF1G, Eukaryotic elongation factor 1 gamma; ER, estrogen receptor; HER‐2, human epidermal growth factor receptor 2; PR, progesterone receptor.

^a^ *p* value from chi‐square test.

### 3.1 Statistical interaction between BMI status and EEF1G expression

During a median follow‐up of 95.5 months, a total of 248 patients experienced disease progression and 165 deaths occurred. In analysis of overall patients, no significant association of EEF1G expression with BC prognosis was found in either univariate or multivariable models. As significant interactions between EEF1G expression and BMI status were observed regarding the OS and PFS (*p*_interaction_ = 0.018 for OS; *p*_interaction_ = 0.010 for PFS), stratification analyses by BMI status were conducted. In patients with BMI ≤ 24 kg/m^2^, higher EEF1G expression was associated with a better prognosis (HR = 0.67, 95% CI: 0.43–1.03 for overall mortality, and 0.60, 95% CI: 0.42–0.86 for progression). Conversely, in patients with BMI > 24 kg/m^2^, higher EEF1G expression was associated with poorer prognosis (HR = 1.74, 95% CI: 0.96–3.17 for overall mortality; HR = 1.63, 95% CI: 1.00–2.66 for progression) (Table 2).

**TABLE 2:** Association of EEF1G expression and BC prognosis by BMI status.

BMI	EEF1G expression	Total (%)	Overall mortality		Disease progression	
			Event	HR (95% CI)^a^	Event	HR (95% CI)^a^
Overall	Low	530 (52.4)	87	1.00 (reference)	134	1.00 (reference)
	High	481 (47.6)	78	0.93 (0.66–1.32)	114	0.86 (0.65–1.14)
≤24	Low	311 (51.7)	56	1.00 (reference)	88	1.00 (reference)
	High	291 (48.3)	42	0.67 (0.43–1.03)	59	**0.60 (0.42–0.86)**
>24	Low	189 (53.5)	27	1.00 (reference)	40	1.00 (reference)
	High	164 (46.5)	30	1.74 (0.96–3.17)	49	**1.63 (1.00–2.66)**
*p*_interaction_				**0.018**		**0.010**

*Note*: Bold indicates statistically significant values.

Abbreviations: BMI, body mass index; EEF1G, Eukaryotic elongation factor 1 gamma.

^a^ Adjusting for age at diagnosis, ER status, HER2, clinical stage, grade.

### 3.2 Differential signaling pathways associated with EEF1G stratified by BMI status

**FIGURE 1:**
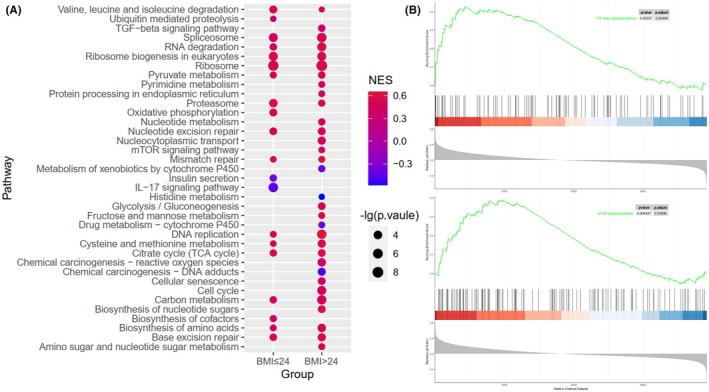
GSEA analysis of GSE78958 dataset. (A) The bubble diagram shows that GSEA results of pathways associated with EEF1G expression in GSE78958 classified by BMI. Bubble size represents the ‐lg of the *p*‐value. Color represents the value of the normalized enrichment score (NES). (B) GSEA analysis shows that EEF1G expression was associated with upregulation of TGF‐β and mTOR signaling pathways in BC patients with BMI > 24 kg/m^2^.

GSEA analysis showed distinct pathway enrichment between different EEF1G expression groups. Predominantly, differentially expressed genes in these groups were enriched in pathways such as ribosome biogenesis in eukaryotes, ribosomes, DNA replication, spliceosome, nucleotide excision repair, and RNA degradation. Furthermore, in patients with BMI > 24 kg/m^2^, high EEF1G expression was associated with the activation of several metabolic pathways, including glycolysis/gluconeogenesis, nucleotide metabolism, fructose and mannose metabolism, pyrimidine metabolism, amino sugar and nucleotide sugar metabolism. Additionally, cancer‐promoting pathways such as mTOR signaling and TGF‐β signaling, were also activated in this group. These association were not statistically significant in patients with a BMI ≤ 24 kg/m^2^ (Figure 1).

### 3.3 Variation in EEF1G‐interacting genes stratified by BMI status

**FIGURE 2:**
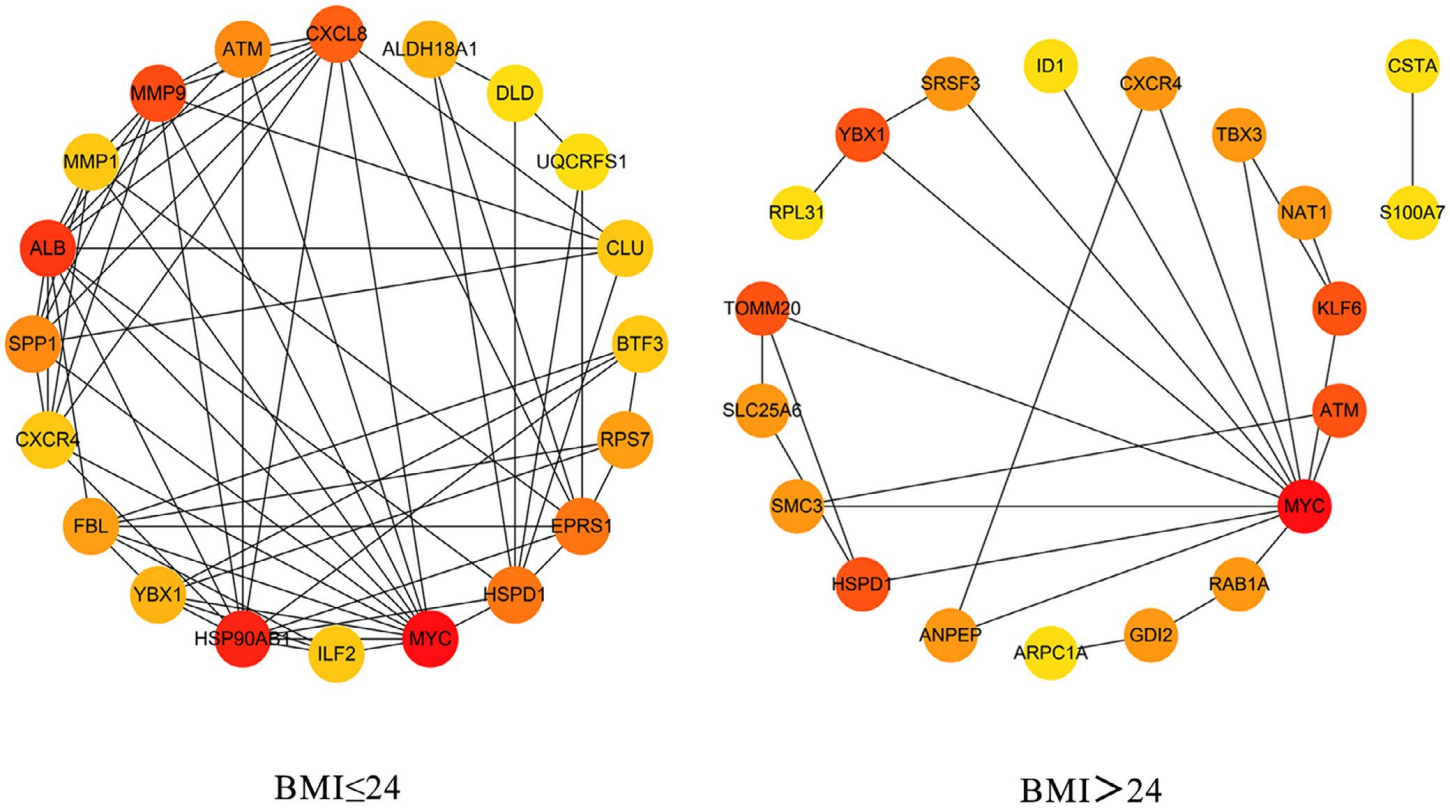
Interaction network diagram of the top 20 key genes associated with EEF1G in different BMI status. The colors represent the intensity of the interaction, with red being the strongest and yellow the weakest. The nodes represent proteins; the edges represent protein interactions.

**FIGURE 3:**
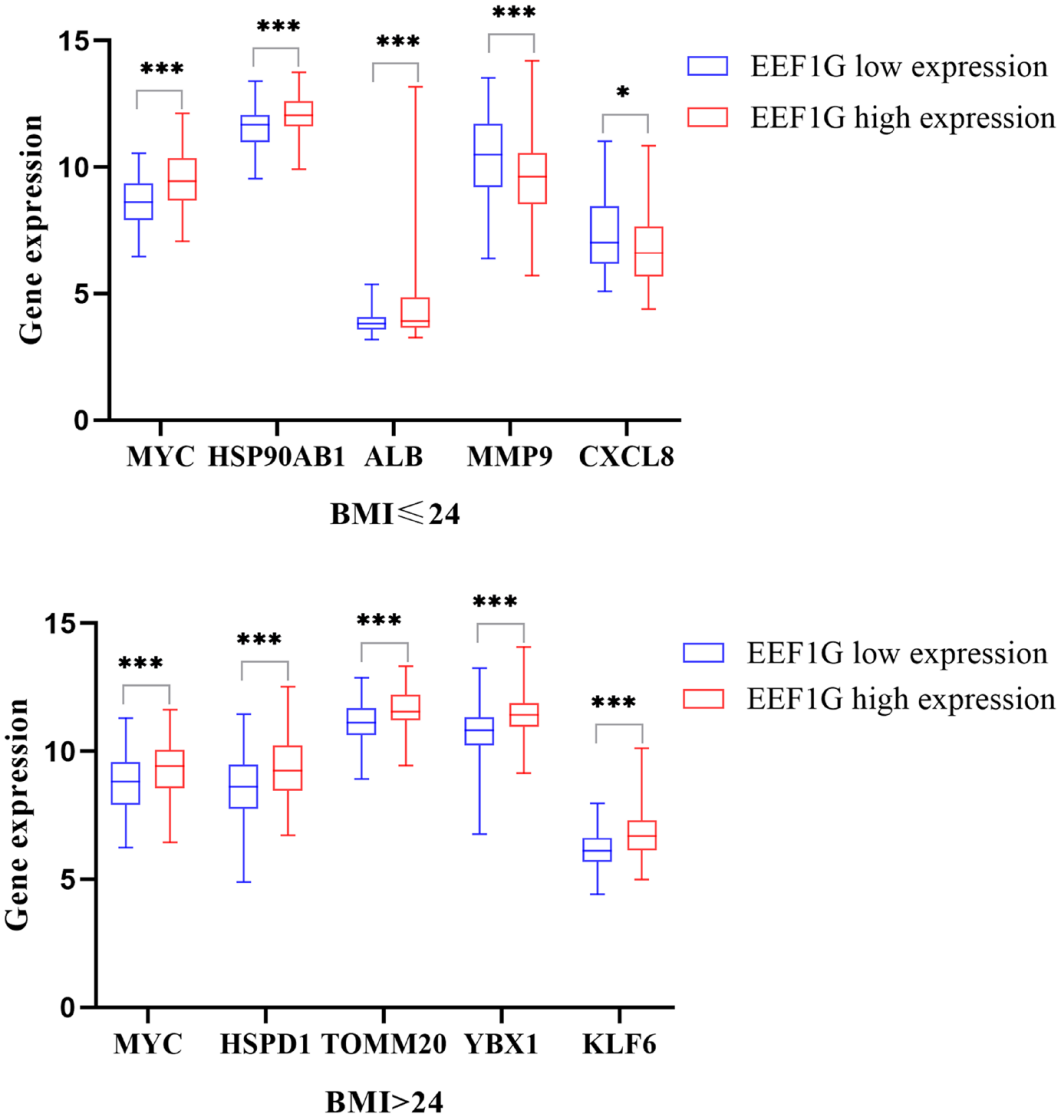
Grouped histograms of the top five genes that interact strongly with EEF1G in different BMI status. *p < 0.05; **p < 0.01; ***p < 0.001.

A PPI network was constructed to identify key genes interacting with EEF1G in BC patients stratified by BMI status (Figure 2). In patients with BMI ≤ 24 kg/m^2^, the top five genes exhibiting the strongest interaction with *EEF1G* were MYC Proto‐Oncogene (*MYC*), Heat Shock Protein 90 Alpha Family Class B Member 1 (*HSP90AB1*), Albumin (*ALB*), Matrix Metallopeptidase 9 (*MMP9*), and C‐X‐C Motif Chemokine Ligand 8 (*CXCL8*). Conversely, in patients with a BMI > 24 kg/m^2^, the top interacting genes identified were *MYC*, Heat Shock Protein Family D (Hsp60) Member 1 (*HSPD1*), Translocase of Outer Mitochondrial Membrane 20 (*TOMM20*), Y‐Box Binding Protein 1 (*YBX1*), and KLF Transcription Factor 6 (*KLF6*). Moreover, correlation analysis showed distinct associations between *EEF1G* expression and these interacting genes in different BMI groups. In patients with a BMI ≤ 24 kg/m^2^, *EEF1G* expression was positively correlated with *MYC*, *HSP90AB1*, *ALB* and negatively correlated with *MMP9* and *CXCL8*. For patients with a BMI > 24 kg/m^2^, *EEF1G* expression showed positive associations with *MYC*, *HSPD1*, *TOMM20*, *YBX1* and *KLF6* (Figure 3).

## 4 DISCUSSION

In this investigation of 1011 BC patients, our analysis revealed a BMI‐dependent prognostic impact of EEF1G expression. Notably, EEF1G expression was associated with improved prognosis in patients with a BMI ≤ 24 kg/m^2^, contrasting an adverse prognosis in those with a BMI > 24 kg/m^2^. These findings underscore the complex interaction between EEF1G expression and metabolic status in BC. The subsequent GSEA analysis on the GSE78958 dataset delineated the distinct pathways influenced by *EEF1G* in varying BMI cohorts, shedding light on the molecular mechanisms that may drive these differential prognostic outcomes. Additionally, the identification of key genes interacting with *EEF1G* across different BMI statuses further emphasizes the nuanced relationship between *EEF1G* expression, the tumor microenvironment, and metabolic factors in BC. Our findings not only highlight the importance of considering BMI in the prognostic evaluation of BC but also suggest potential BMI‐specific therapeutic targets related to EEF1G and its associated molecular pathways.

Adipose tissue, as a major component of the mammary gland, plays a critical role in the communication of the breast microenvironment.^17^ Adipokines secreted by adipocytes are key mediators of adipose signaling pathways in BC.^18^ For example, adipokines such as leptin,^19^ adiponectin,^20^ and resistin^21^ bind to receptors on BC cells, activating various downstream signaling pathways that regulate processes such as cancer cell proliferation, angiogenesis, and apoptosis. Additionally, various cytokines and chemokines secreted by adipose tissue construct the immune microenvironment of BC cells, significantly influencing their apoptosis and migration.^22^, ^23^, ^24^ Breast fat tissue also secretes aromatase, which synthesizes androgens into estrogens,^25^ and serves as a source of free fatty acids and cholesterol, providing ATP for the increased proliferation and energy requirements of cancer cells.^26^ Therefore, the interplay between mammary fat and BC cells is crucial for BC growth and proliferation.

In this context, breast adipose tissue secretion can alter the expression profile of BC cells. For example, when BC cells are cultured in conditioned media from adipose tissue explants from BC patients, there is an increase in the expression of *CD44*, *ADAMTS1* and *adipoR1* in BC cells, leading to increased proliferation, adhesion, and migration.^27^ The secretome profile of adipose tissue is also altered obesity, resulting in changes in the expression profile of BC cells. Hence, the expression and function of the same gene in BC cells may vary under different BMI statuses. EEF1G, beyond its conventional role in protein translation, plays a key role in the cellular stress response and is particularly responsive to changes in the tumor microenvironment.^28^ This suggests that EEF1G may mediate signaling crosstalk between adipocytes and BC cells, contributing to the observed divergent prognostic outcomes between obese and lean BC patients.

We used GSEA to compare the role of EEF1G in BC cells across different BMI status. In BC patients with a BMI ≤ 24 kg/m^2^, EEF1G primarily supports normal cell cycle progression, DNA replication, and transcription and translation processes. In addition, EEF1G is also associated with the functions of spliceosome, mismatch repair, and base excision repair, which are essential for inhibiting uncontrolled cell proliferation and reduce the likelihood of gene mutations in BC cells, contributing to a favorable prognosis. Conversely, in patients with BMI > 24 kg/m^2^, EEF1G was associated with metabolic reprogramming and the activation of oncogenic pathways, potentially leading to adverse outcomes. Metabolic reprogramming is recognized as a hallmark of cancer, promoting the proliferation and survival of cancer cells even under adverse conditions.^29^, ^30^, ^31^ Additionally, our findings align with studies emphasizing the critical role of TGF‐β^32^, ^33^ and mTOR^34^, ^35^, ^36^ signaling pathways in BC progression. TGF‐β, known for its dual role in cancer, initially suppresses tumorigenesis through cytostatic effects and apoptosis.^37^ However, as the tumor progression, TGF‐β shifts to promote tumor metastasis by enhancing the motility and invasiveness of cancer cells.^38^ The mTOR pathway, frequently hyperactivated various cancers, including BC, drives excessive cell proliferation and resistance to apoptosis.^39^ Notably, mTOR signaling is not only pivotal in BC,^35^ but also plays a significant role in obesity development.^40^

Our construction of PPI networks identified several key genes that shed light on the relationship between *EEF1G* expression and BMI status in BC prognosis. Among these genes, *MYC* exhibited the strongest interactions with *EEF1G* across the BMI categories. *MYC* protein is known to modulate a range of cellular processes akin to those implicated in our GSEA results, including cell growth, cell cycle, differentiation, apoptosis, angiogenesis, metabolism, DNA repair, and protein translation.^41^, ^42^ Remarkably, in patients with BMI ≤ 24 kg/m^2^, *EEF1G* interaction suggest a tilt towards favorable BC prognosis. Marked by the association with tumor suppressor protein *HSP90AB1* ^43^ and reduced expression of the BC risk prognostic markers *MMP9* ^44^ and *CXCL8*.^45^ Conversely, in patients with BMI > 24 kg/m^2^, *EEF1G* positively associated with key genes (*HSPD1*,^46^ *YBX1*,^47^ and *KLF6* ^48^), which have been shown to promote BC recurrence and metastasis.

As BC is a hormone‐dependent tumor, hormone receptor status plays a crucial role in its prognosis.^49^ Exploring the interaction between EEF1G and hormone receptors helps to provide significant clues for personalized treatment of BC. However, we did not observe a differential prognostic impact of EEF1G based on ER/PR/HER2 status (Table S2). This suggests that the prognostic value of EEF1G may be independent of hormone receptor status. Further studies are warranted to fully elucidate the potential interactions and their implications.

Given the emerging interest in eukaryotic elongation factor like EEF2K and EEF1A1 as potential therapeutic targets,^50^, ^51^ our findings propose EEF1G as a novel candidate. While mTOR and TGF‐β signaling inhibitors and drugs targeting metabolic pathways have shown promise in BC therapy,^52^, ^53^, ^54^, ^55^, ^56^ their efficacy often encounters challenges such as tumor heterogeneity.^29^ This underscores the need for reliable biomarkers to refine patient selection for precision therapy. Our study thus advocates for exploring EEF1G‐targeted treatments to potentially enhance BC survival outcomes.

Our study has several limitations that should be acknowledged. Firstly, the absence of public databases containing both BMI and outcome information restricted our ability to directly corroborate our findings. However, the observed differences in EEF1G‐related pathways and gene interactions across BMI categories provide indirect validation. Secondly, the inclusion of patients with tumors larger than 1 cm could introduce selective bias. Yet, the consistence in EEF1G expression independent of tumor size suggests minimal impact on the observed EEF1G‐prognosis association. Thirdly, the lack of analysis on the specific roles of different BC subtypes, partially because the sample size for each subtype might not have been sufficient to draw definitive conclusions. Future studies investigating the subtype‐specific implications of EEF1G with larger sample sizes to provide a more comprehensive understanding of its prognostic value are warranted. Lastly, although we adjusted for known clinicopathological features, the lack of comprehensive treatment data and comorbidity information could have introduced confounding factors. Future studies including the incidence of comorbidities such as diabetes to better evaluate the prognostic value of EEF1G in BC are needed.

## 5 CONCLUSION

This study provides novel insights into the role of EEF1G in BC prognosis, revealing its dual function as a protective factor in patients with a BMI ≤ 24 kg/m^2^ and as a risk factor in patients with a BMI > 24 kg/m^2^. Analysis of the GSE78958 dataset further elucidated the differential biological functions of EEF1G across varying BMI status, reinforcing the validity of our results. These findings contribute to the understanding of EEF1G in BC prognosis and underscore its potential as both a prognostic marker and a promising target for therapeutic intervention in BC patients, contingent on the BMI status.

## AUTHOR CONTRIBUTIONS

**Na Li:** Data curation (equal); funding acquisition (equal); investigation (equal); resources (equal). **Chengkun Xiao:** Conceptualization (lead); formal analysis (lead); software (equal); writing – original draft (lead). **Shushu Han:** Data curation (equal); investigation (equal); resources (equal). **Minjie Lu:** Data curation (equal); investigation (equal). **Qianxin Chen:** Data curation (equal); funding acquisition (equal). **Yuanzhong Yang:** Data curation (equal); resources (equal). **Luying Tang:** Data curation (equal); resources (equal). **Zefang Ren:** Project administration (lead); supervision (equal); validation (equal); writing – review and editing (equal). **Lin Xu:** Validation (equal); visualization (equal); writing – review and editing (equal).

## FUNDING INFORMATION

This study was funded by the Natural Science Foundation of China (No. 82373661; No. 81973115) & the Science and Technology Planning Project of Guangdong Province, China.

## CONFLICT OF INTEREST STATEMENT

The authors report there are no competing interests to declare.

## Supporting information


Appendix S1.

## Data Availability

The datasets used and/or analyzed during the current study are available from the corresponding author on reasonable request.
