# Supplementary material for: BMI‐dependent prognostic role of EEF1G in breast cancer: A 15‐year follow‐up of the Guangzhou Breast Cancer Cohort Study
Source: Cancer Med. 2025 Sep 9;14(17):e70227. doi: 10.1002/cam4.70227 (PMC12420659; doi:10.1002/cam4.70227)
Supplement: Supplementary file 1 — Appendix S1. [file CAM4-14-e70227-s001.docx]

# Supplementary Material

Supplementary table 1. Demographic and clinicopathological characteristics and the associations with EEF1G mRNA expression in GSE78958.

Supplementary table 2. Association of EEF1G expression and BC prognosis by hormone receptor status.

Supplementary figure 1. Ridge map of GSEA results in BC patients with BMI≤24.

Supplementary figure 2. Ridge map of GSEA results in BC patients with BMI＞24.

Supplementary figure 3. The top three signaling pathways most strongly associated with EEF1G in BC patients with BMI≤24.

Supplementary figure 4. The top three signaling pathways most strongly associated with EEF1G in BC patients with BMI＞24.

Supplementary figure 5. Correlation between EEF1G expression and immune cell infiltration in BC patients with BMI<24 kg/m^2^.

Supplementary figure 6. Correlation between EEF1G expression and immune cell infiltration in BC patients with BMI≥24 kg/m^2^.

| Supplementary table 1. Demographic and clinicopathological characteristics and the associations with EEF1G mRNA expression in GSE78958 | | | | |
| --- | --- | --- | --- | --- |
| Characteristics | *N* (%) | Low expression  (n=202) | High expression  (n=202) | *p* value ^a^ |
| Ethnicity |  |  |  | 0.209 |
| European American | 288 (71.3) | 148 (73.3) | 140 (69.3) |  |
| African American | 94 (23.3) | 47 (23.3) | 47 (23.3) |  |
| Other | 22 (5.4) | 7 (3.5) | 15 (7.4) |  |
| Missing | 0 |  |  |  |
| BMI |  |  |  | 0.915 |
| ≤25 | 131 (32.4) | 66 (32.7) | 65 (32.2) |  |
| ＞25 | 273 (67.6) | 136 (67.3) | 137 (67.8) |  |
| Missing | 0 |  |  |  |
| Grade |  |  |  | **0.043** |
| Low | 83 (20.6) | 33 (16.4) | 50 (24.8) |  |
| Moderate | 146 (36.2) | 83 (41.3) | 63 (31.2) |  |
| High | 174 (43.2) | 85 (42.3) | 89 (44.1) |  |
| Missing | 1 |  |  |  |
| Tumor subtype |  |  |  | 0.924 |
| Luminal A | 209 (52.5) | 106 (53.8) | 103 (51.2） |  |
| Luminal B | 43 (10.8) | 22 (11.2) | 21 (10.4) |  |
| HER2 riched | 48 (12.1) | 23 (11.7) | 25 (12.4) |  |
| Basal like | 98 (24.6) | 46 (23.4) | 52 (25.9) |  |
| Missing | 6 |  |  |  |
| Clinical stage |  |  |  | 0.854 |
| Ⅰ/Ⅱ | 332 (82.2) | 164 (83.7) | 168 (80.8) |  |
| Ⅲ/Ⅳ | 72 (17.8) | 32 (16.3) | 40 (19.2) |  |
| Missing | 0 |  |  |  |
| ^a^ *p* value for chi-square test | | | | |

| Supplementary table 2. Association of EEF1G expression and BC prognosis by hormone receptor status | | | | | | | | |
| --- | --- | --- | --- | --- | --- | --- | --- | --- |
|  | EEF1G expression | Total (%) | Overall mortality | |  | | Disease progression | |
|  |  |  | Event | HR (95% CI)^a^ | | Event | | HR (95% CI)^a^ |
| **ER** |  | | | | | | | |
| Negative | Low | 117 (45.5) | 21 | 1.00 (reference) | | 35 | | 1.00 (reference) |
|  | High | 140 (54.5) | 30 | 0.97 (0.54-1.74) | | 37 | | 0.68 (0.42-1.12) |
|  |  |  |  |  | |  | |  |
| Positive | Low | 386 (54.8) | 58 | 1.00 (reference) | | 91 | | 1.00 (reference) |
|  | High | 319 (45.2) | 46 | 0.91 (0.60-1.39) | | 73 | | 0.94 (0.67-1.31) |
| *P*_interaction_ |  |  |  | 0.85 | |  | | 0.26 |
|  |  |  |  |  | |  | |  |
| **PR** |  |  |  |  | |  | |  |
| Negative | Low | 137 (52.3) | 24 | 1.00 (reference) | | 35 | | 1.00 (reference) |
|  | High | 125 (47.7) | 25 | 094 (0.50-1.77) | | 33 | | 0.91 (0.63-1.55) |
|  |  |  |  |  | |  | |  |
| Positive | Low | 364 (52.1) | 54 | 1.00 (reference) | | 90 | | 1.00 (reference) |
|  | High | 334 (47.9) | 52 | 0.62 (0.35-1.09) | | 77 | | 0.78 (0.55-1.10) |
| *P*_interaction_ |  |  |  | 0.94 | |  | | 0.63 |
|  |  |  |  |  | |  | |  |
| **HER-2** |  |  |  |  | |  | |  |
| Negative | Low | 354 (52.1) | 56 | 1.00 (reference) | | 88 | | 1.00 (reference) |
|  | High | 325 (47.9) | 54 | 1.04 (0.68-1.58) | | 84 | | 0.92 (0.66-1.29) |
|  |  |  |  |  | |  | |  |
| Equivocal | Low | 46 (54.1) | 12 | 1.00 (reference) | | 17 | | 1.00 (reference) |
|  | High | 39 (45.9) | 6 | 0.49 (0.11-1.30) | | 7 | | 0.36 (0.13-1.03) |
|  |  |  |  |  | |  | |  |
| Positive | Low | 130 (52.6) | 19 | 1.00 (reference) | | 29 | | 1.00 (reference) |
|  | High | 117 (47.4) | 18 | 0.98 (0.49-1.98) | | 23 | | 0.71 (0.38-1.33) |
| *P*_interaction_^b^ |  |  |  | 0.17 | |  | | 0.10 |
| *P*_interaction_^c^ |  |  |  | 0.51 | |  | | 0.48 |
| ^a^: Adjusting for age at diagnosis, ER status, HER-2 status, clinical stage, grade.  ^b^: Refers to the value of the interaction between equivocal and negative.  ^c^: Refers to the value of the interaction between positive and negative.  EEF1G: Eukaryotic elongation factor 1 gamma. | | | | | | | | |


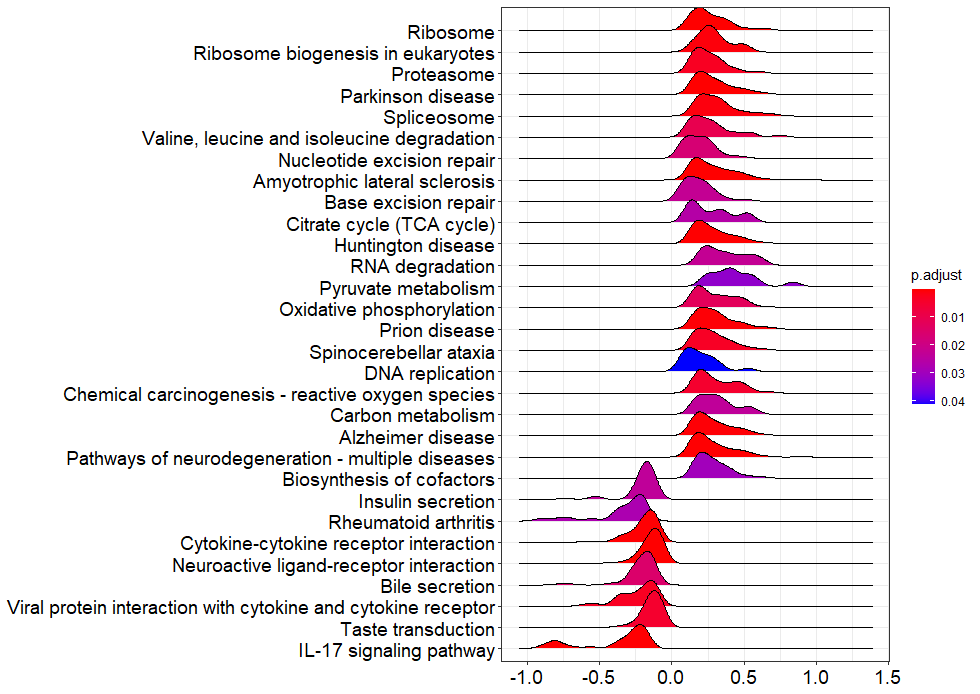


Supplementary figure 1. Ridge map of GSEA results in BC patients with BMI≤24.

.


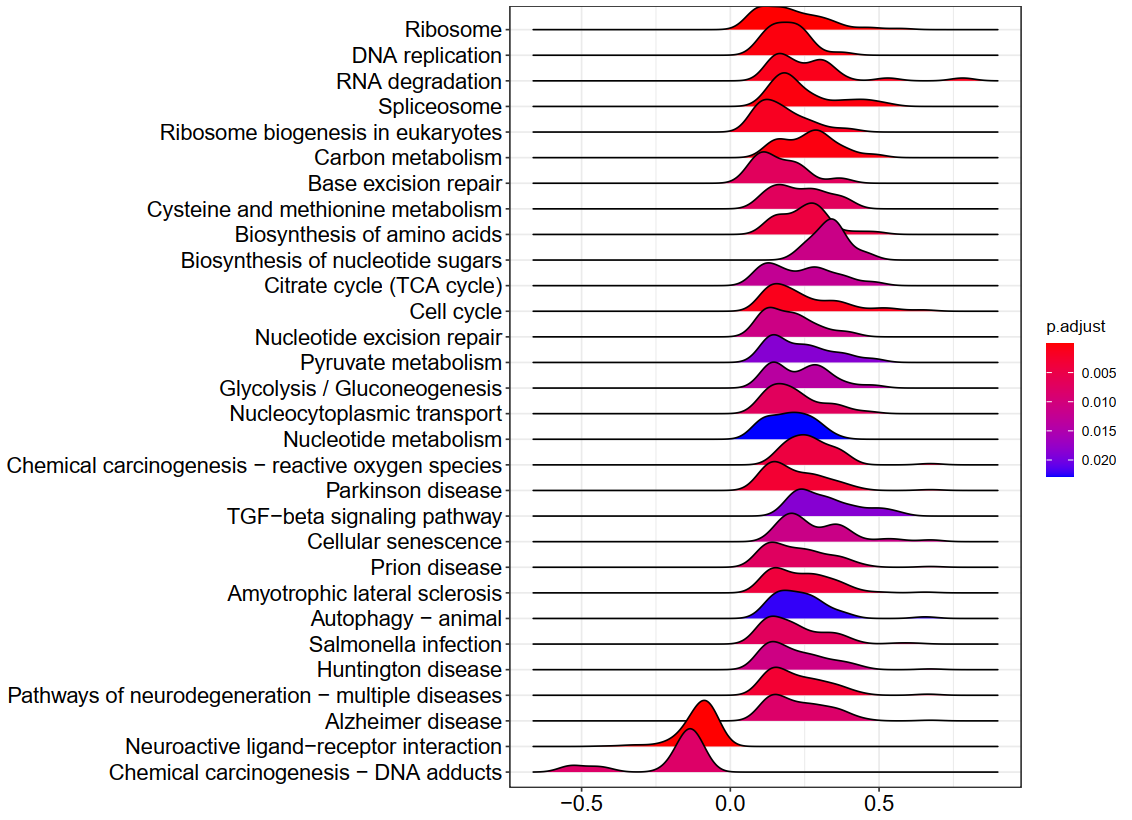


Supplementary figure 2. Ridge map of GSEA results in BC patients with BMI＞24.


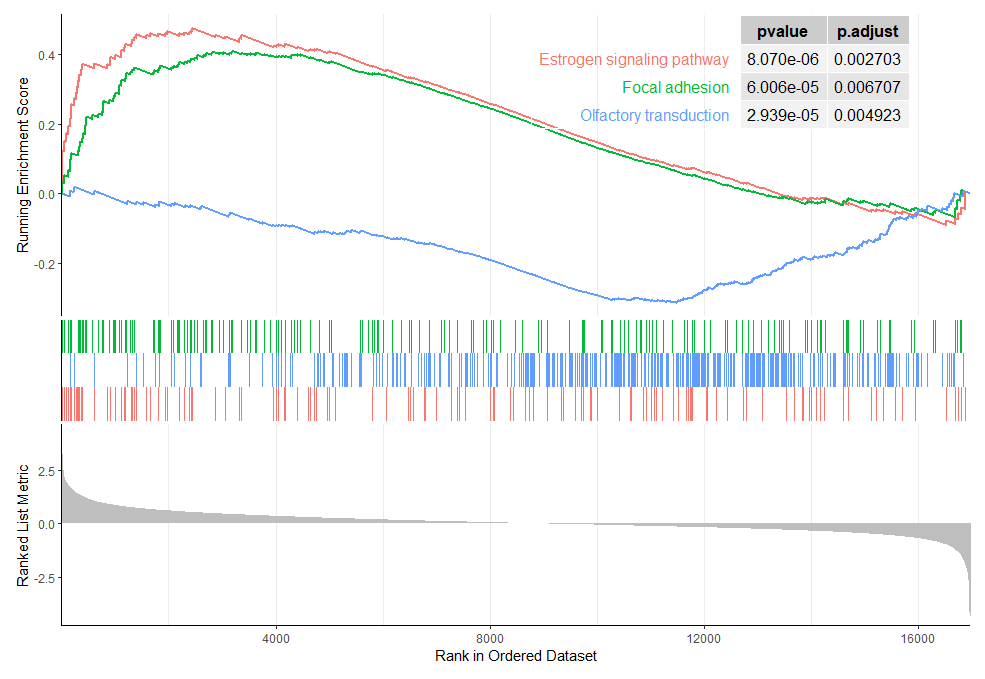
 Supplementary figure 3. The top three signaling pathways most strongly associated with EEF1G in BC patients with BMI≤24.


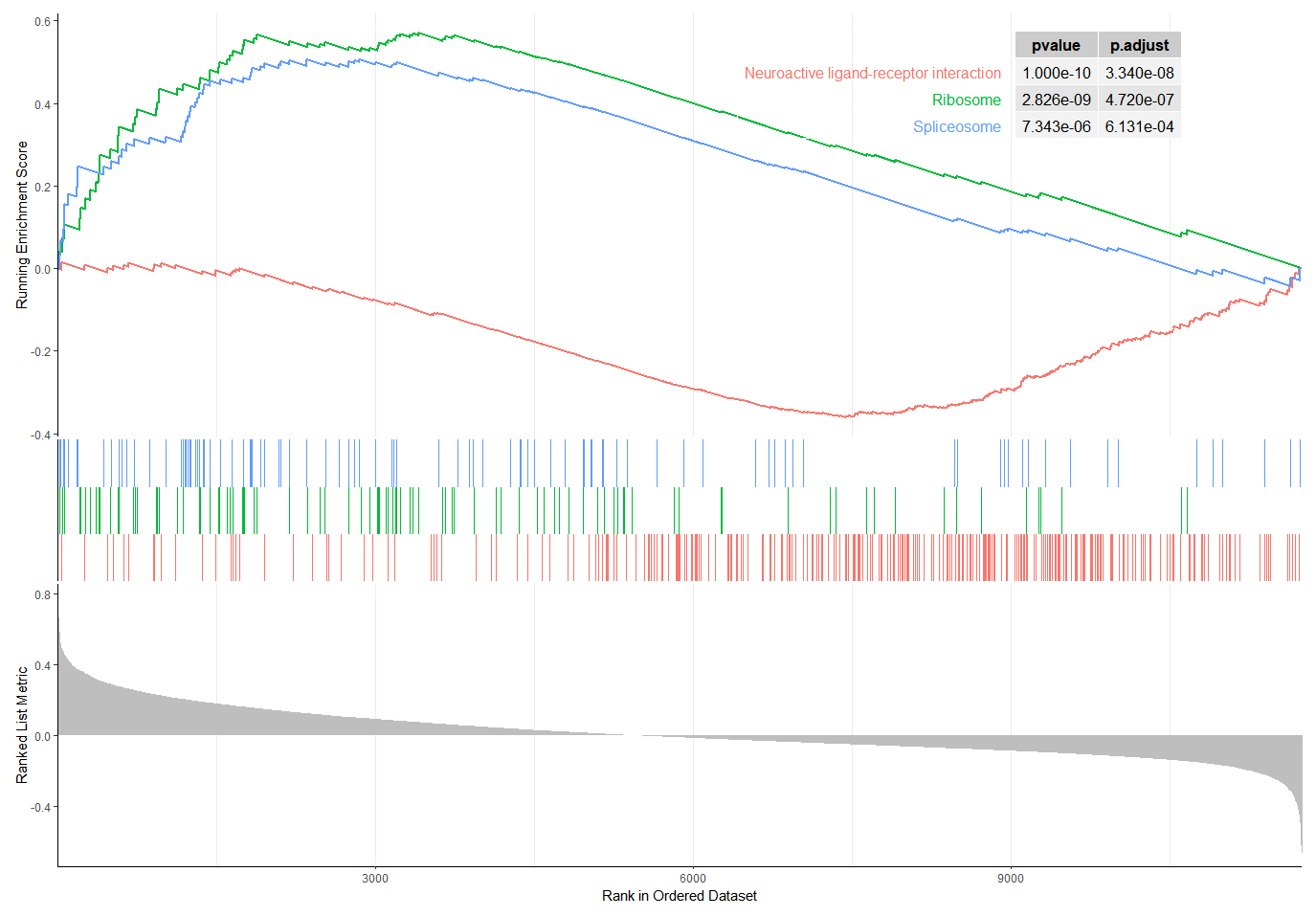


Supplementary figure 4. The top three signaling pathways most strongly associated with EEF1G in BC patients with BMI＞24.


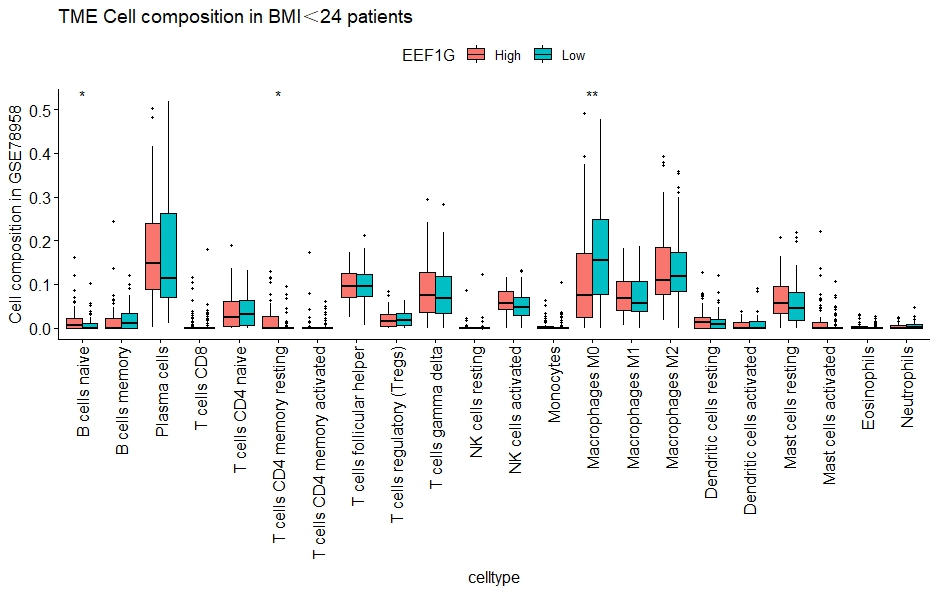


Supplementary figure 5. Correlation between EEF1G expression and immune cell infiltration in BC patients with BMI<24 kg/m^2^.


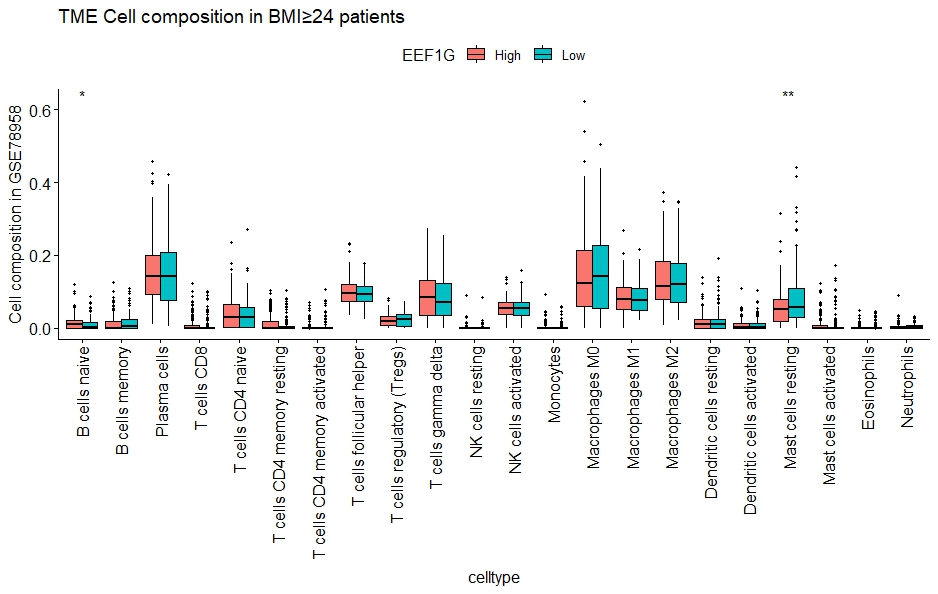


Supplementary figure 6. Correlation between EEF1G expression and immune cell infiltration in BC patients with BMI≥24 kg/m^2^.
